# A Low-Cycle Fatigue Life Prediction Method for a Drive Shaft Considering the Effects of Loading and Strength Degradation

**DOI:** 10.3390/ma19102164

**Published:** 2026-05-21

**Authors:** Li Yang, Xingsheng Yu, Feng Liu, Liyong Wang, Jinle Zhang, Ximing Zhang, Jing Zhang

**Affiliations:** 1 Beijing Petroleum Machinery Co., Ltd., Beijing 102206, China; 2 The Ministry of Education Key Laboratory of Modem Measurement and Control Technology, Beijing Information Science & Technology University, Beijing 100192, China; 3 China North Vehicle Research Institute, Beijing 100072, China

**Keywords:** fatigue strength degradation, cumulative damage, life prediction

## Abstract

The low-cycle fatigue failure of drive shafts under complex service conditions constitutes a critical issue that undermines the structural integrity and service safety of the transmission system in special vehicles. To improve the prediction accuracy of the low-cycle fatigue life of drive shafts, a low-cycle fatigue life prediction method for the drive shaft that accounts for load effects and strength degradation is proposed. A fatigue life prediction model that accounts for the mean stress effect and fatigue strength degradation is proposed by introducing dynamically degrading fatigue strength into the mean stress-refined SWT (Smith–Watson–Topper) model. A fatigue cumulative damage model that considers load interactions and fatigue strength degradation is also proposed, in which the load ratio is introduced to quantitatively describe the extent of the influence of load interactions on the damage process. Furthermore, the dynamically degrading fatigue strength is incorporated into the M-H (Manson–Halford) model. Finally, the stress–strain responses at the critical locations of the drive shaft are analyzed using the finite element model, and the fatigue life of the drive shaft under the load spectrum is calculated using the improved fatigue life prediction model and the improved fatigue cumulative damage model. The results indicate that the improved life prediction method, which considers load effects and strength degradation, can effectively enhance the accuracy of fatigue life prediction for the drive shaft.

## 1. Introduction

The drive shaft is a critical component in the integrated transmission system of tracked vehicles, responsible for transmitting power and motion. Its structure features local sharp notches and geometric discontinuities, which lead to significant macroscopic plastic deformation in hazardous zones. Consequently, fatigue damage occurs under a relatively low number of loading cycles [1]. The drive shaft serves as a pivotal component within the integrated transmission system of tracked vehicles, tasked with transmitting power and motion. Due to the presence of local sharp notches and geometric discontinuities in its structure, pronounced macroscopic plastic deformation occurs in critical regions. As a result, fatigue damage manifests at a relatively low number of cycles [2,3]. During its service, the drive shaft is subjected to complex and high-level alternating loads. The amplitude of the maximum stress and the mean stress significantly impact the fatigue life of the metallic material. Moreover, the stress levels in preceding and subsequent loading, as well as the loading sequence, are closely related to the calculation of fatigue cumulative damage [4]. Moreover, traditional low-cycle fatigue life prediction methods seldom account for the influence of fatigue strength degradation [5]. The aforementioned various influencing factors render most existing fatigue life prediction methods inadequate for meeting practical engineering requirements [6,7,8,9]. Consequently, there is an urgent need to propose a low-cycle fatigue life prediction method for drive shafts that takes into account the effects of special loading and strength degradation. This is of great significance for ensuring the normal, stable, and safe operation of special vehicles.

Engineering demands serve as a powerful driving force for fatigue research. Currently, a vast number of experts and scholars in this field have proposed a wide variety of fatigue life prediction methods. Regarding the modification and improvement of low-cycle fatigue prediction models, Wang et al. [10] established a relationship between the Walker exponent and the ultimate strength of the material and introduced the Walker exponent to enhance the strain-life model based on the M-C (Mason–Coffin) model that accounts for the mean stress effect. Wu et al. [11] proposed an improved Gerber life prediction model considering the influence of mean stress and combined it with finite element simulation to establish a fatigue life prediction method for turbine shafts. Liu et al. [12] introduced a fatigue strength correction factor β to modify the M-C formula, establishing a low-cycle fatigue life prediction model that accounts for the impact of thermal aging on fatigue life. Through comparison, it is found that the improved model exhibited better predictive performance. Yang et al. [13] proposed a low-cycle fatigue life prediction method for turbine disks that considers the mean stress effect and derived a corrected fatigue life prediction model for turbine disks based on the Walker criterion. Shi et al. [14] took into account the influence of material hardening behavior under asymmetric cyclic loading on the low-cycle fatigue of welded structures and proposed a numerical structural strain method that considers linear hardening behavior. Rwnzo et al. [15,16,17] conducted a series of multiaxial fatigue behavior investigations via extensive experiments and proposed various modified damage models with superior prediction accuracy compared with conventional models, providing technical insights and data support for the research on damage failure under different fatigue failure modes. They also analyzed the impact of the Bauschinger effect on the low-cycle fatigue of welded structures under symmetric cyclic loading. The existing improved models only take into account the influence of a single factor, such as load effect, strength degradation, or cyclic material characteristics, and the accuracy of life prediction still needs to be improved.

In addition, during the service life of engineering structural components, due to the combined effects of multiple factors such as their operating environment, manufacturing and processing defects, and complex loading conditions, it is inadequate to merely consider cumulative damage as a linear summation of individual damage instances. The loading sequence and interactions cannot be overlooked, and the precise quantitative characterization of fatigue cumulative damage has long been recognized as a challenging problem. Zhou et al. [18] took into account the internal material damage and irreversible degradation caused by cyclic loading in engineering structural components, introduced a material memory property function, and established a novel fatigue cumulative damage model. Li et al. [19] proposed an improved M-H (Manson–Halford) nonlinear cumulative damage model to address the inaccuracy in damage analysis of the traditional M-H model. They drew an analogy between the decomposition of organic matter in ecology and the degradation of mechanical properties of materials under multilevel loading, thereby formulating the enhanced model. Zhao and Gao [20,21] analyzed the causes of prediction errors in traditional fatigue cumulative damage models, taking into account the effects of load interaction and strength degradation on fatigue cumulative damage. They conducted improved research work based on the M-H model. However, most of these models merely reflect the influence of load interaction on fatigue cumulative damage by introducing the stress ratio between two consecutive load levels or simplifying the fatigue strength degradation mechanism as a linear degradation with the number of cycles, which makes it difficult to meet the practical engineering requirements.

In response to the aforementioned issues, this study proposes a low-cycle fatigue life prediction method for drive shafts that accounts for load effects and strength degradation. Monotonic torsion tests and low-cycle torsional fatigue tests are conducted on the drive shaft to obtain monotonic torsional stress–strain curves and cyclic torsional stable hysteresis curves. The cyclic mechanical properties under different strain amplitudes are analyzed, and a finite element model of the drive shaft that considers the material’s cyclic characteristics is established. A fatigue life prediction model that accounts for the mean stress effect and fatigue strength degradation is proposed by introducing fatigue strength that degrades with increasing cycle numbers into the Walker exponent of the SWT model. Additionally, a fatigue cumulative damage model that considers load interactions and fatigue strength degradation is proposed, in which the load ratio is introduced to modify the life ratio characteristic of the M-H model, quantitatively describing the extent of the influence of load interactions on the damage process, and dynamically degrading fatigue strength is further incorporated into the model. Ultimately, the finite element model of the drive shaft is employed to analyze the stress–strain responses at critical locations of the drive shaft. The fatigue life under single-level stress is obtained by combining the improved fatigue life prediction model, and then the fatigue life of the drive shaft under the load spectrum is calculated using the improved fatigue cumulative damage model.

## 2. Fatigue Torsion Test of Drive Shaft

Firstly, monotonic torsion tests are conducted on the drive shaft to obtain the monotonic torsion stress–strain curve. Subsequently, low-cycle torsional fatigue tests are performed under different strain amplitudes to acquire the cyclic stable hysteresis loops and fatigue life. The cyclic mechanical characteristics under various strain amplitudes are analyzed to provide material properties and model validation data for subsequent finite element analysis.

### 2.1. Test Sample

**Figure 1:**
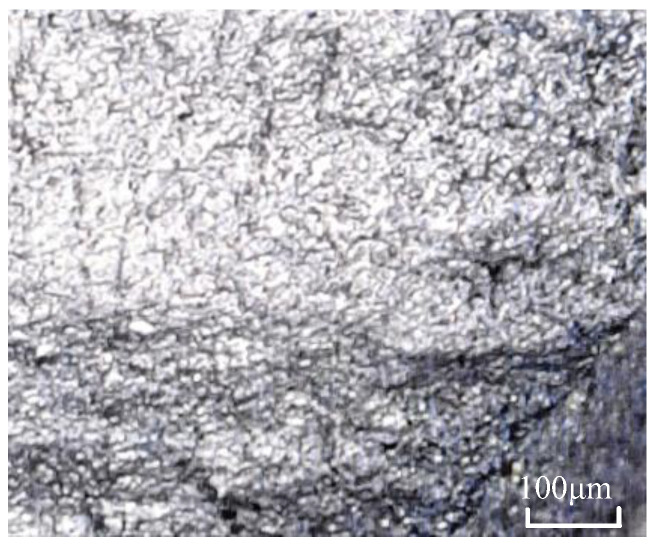
Metallographic structure of 300M steel.

The test material for the drive shaft of special vehicles is 40CrNi2Si2MoVA (hereinafter referred to as 300M). It is commonly used as the primary load-bearing component in structures. Its chemical composition is shown in Table 1, with relatively high contents of Cr, Mn, Ni, and Si. The heat treatment process is as follows: the specimen is heated in a furnace to 870 °C, held for 1 h for solution treatment, and then quenched in oil. Subsequently, it is reheated in the furnace to 300 °C, held for 2 h for tempering at a moderate temperature, and finally cooled in air. After being ground, polished, and etched, the 300M steel specimen is rinsed with alcohol and air-dried. The microstructural morphology is shown in Figure 1. The results indicate that the microstructure primarily consists of lath martensite, with some lower bainite and a small amount of austenite retained. The relevant data are sourced from the China Aeronautical Materials Handbook, the Material Data Handbook for Aeroengine Design, and material data provided by a certain organization.

**Figure 2:**
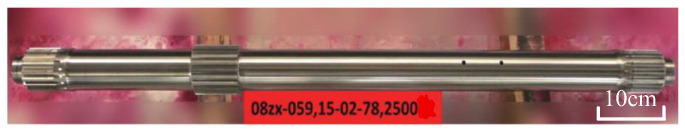
Test sample.

The drive shaft obtains a stable working environment and transmits working loads by connecting with surrounding components. The test specimen of the drive shaft is shown in Figure 2. To meet the requirements of overall structural design and assembly, the drive shaft features a relatively complex structure, incorporating intricate geometric shapes such as relief grooves and splines on the shaft. Meanwhile, these areas often lead to abrupt changes in the cross-sectional shape of the drive shaft, causing significant stress concentrations, which in turn can result in fatigue failure in certain localized regions due to high cyclic stresses.

### 2.2. Test Scheme

**Figure 3:**
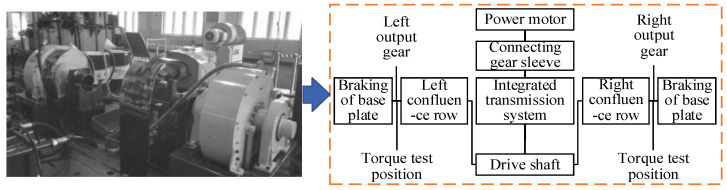
Test bench.

The integrated transmission system test bench is constructed as shown in Figure 3. The output gears of the busbars on both sides of the integrated transmission system are fixed onto the base plate, and the torques of these output gears on both sides are collected in real time. The intermediate involute spline on the drive shaft of the integrated transmission system is connected to the transmission mechanism to serve as the input, while the involute splines at both ends of the drive shaft are connected to the busbars to act as the outputs. Due to structural layout and spatial constraints within the transmission system, it is difficult to directly measure the torque of the main shaft. Therefore, an indirect measurement approach is adopted by measuring the torque of the output gears of the busbars, which are connected to the output splines of the drive shaft.

The experimental environment is a laboratory setting with a temperature of 20 °C. Initially, a monotonic torsion test is conducted on the drive shaft. The specimen is loaded using displacement control at a loading rate of 0.02°/s. As the loading angle increased, the torque borne by the specimen gradually rose, accompanied by a corresponding increase in torsional deformation. When the drive shaft underwent buckling deformation and lost its ability to resist torsional deformation, the specimen is deemed to have failed. The basic procedure for the low-cycle torsional fatigue test is consistent with that of the monotonic torsion test. During the phase from the start of test loading to the achievement of cyclic stability, loading is primarily controlled by strain. The response of an extensometer is utilized to ensure that the specimen attains a constant strain amplitude throughout the test. The extensometer is a strain-measuring device that directly acquires the torsional shear strain of the drive shaft, while the torque is independently measured by the torque sensor of the test bench. The measured strain and torque data are, respectively, adopted for the calculation of torsional shear stress in different deformation stages. The extensometer is mounted on the smooth cylindrical section of the shorter shaft segment of the test specimen and arranged as close as possible to the spline at the shaft end. The contact area between the extensometer and the specimen is polished to a smooth finish, and the extensometer is circumferentially clamped and precisely aligned to ensure that its measuring direction is consistent with the torsional deformation direction of the specimen without any deviation. According to the previous simulation analysis by the research team, the transition zone between the spline root and tool withdrawal groove at the shorter shaft end of the test specimen is the fatigue life-critical region of the drive shaft. Strain gauges were affixed at this position, with one strain gauge arranged every three teeth along the circumferential direction. A total of seven strain gauges were installed, and the average value of the measured strain data was adopted for subsequent analysis.

The loading waveform is a sinusoidal triangular wave with a frequency ranging from 0.1 Hz to 0.5 Hz and a strain ratio of R = −1. Considering test costs and fitting accuracy, low-cycle fatigue tests are conducted using three levels of strain amplitudes: 0.3%, 0.6%, and 0.9%, with at least three specimens tested at each amplitude level. Given the difficulty in measuring the real-time crack length of specimens with existing observational equipment, this study employs an empirical method outlined in the relevant standard [22]. Specifically, the low-cycle torsional fatigue crack initiation life of the component is estimated when the maximum shear stress in the cyclic shear stress-shear strain curve drops below 5% of the peak value of the stabilized hysteresis loop.

### 2.3. Test Results

**Figure 4:**
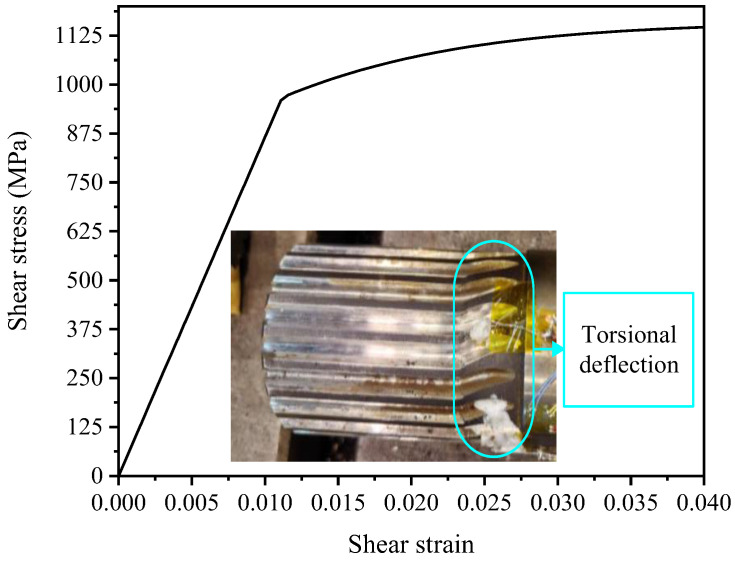
Measured curve.

During the monotonic torsion test, due to the excellent toughness of 300M steel, no obvious visible fracture cracks are observed on the specimen surface. Instead, severe and irreversible torsional buckling deformation occurred when the load exceeded the material’s shear strength limit, as shown in Figure 4. The curves presented in Figure 4 are directly plotted using the experimentally measured torque and shear strain data obtained from the smooth cylindrical section of the short shaft end near the end spline, with no modification or manual adjustment to the raw data values. The data are post-processed using Origin 2018 software to convert the measured torque and angular displacement into shear stress and shear strain, respectively. A simple low-pass filter was applied to remove minor numerical noise, which is a standard practice in experimental mechanics to improve the clarity of the monotonic torsional shear stress–strain curve. At room temperature, 300M steel did not exhibit a distinct yield plane. In this study, the shear stress corresponding to a residual shear strain of 0.3% is defined as the shear yield stress, denoted as *τ*_0.3_. The maximum shear stress on the curve is taken as the ultimate shear strength of the material, denoted as *τ*_b_. The shear modulus *G* of 300M steel at room temperature is determined by calculating the slope of the elastic portion of the monotonic curve.

**Figure 5:**
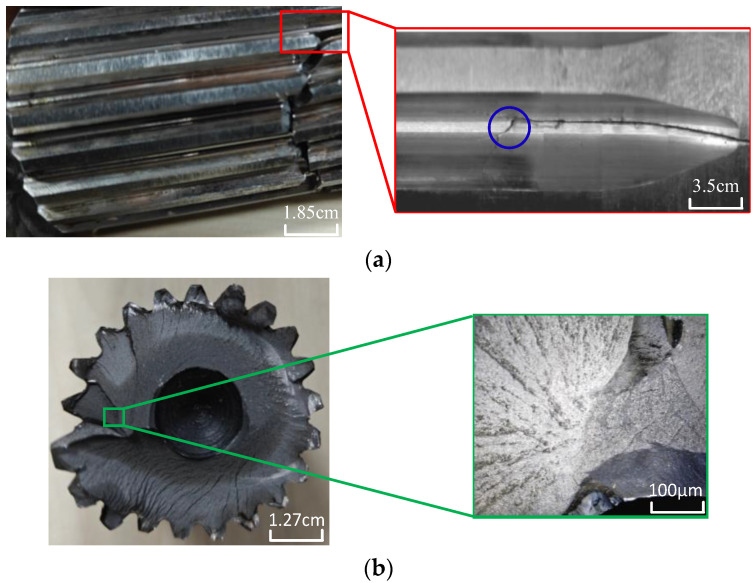
Fracture appearance. (**a**) Fracture sample and crack initiation position. (**b**) Crack initiation position and microscopic appearance.

Low-cycle torsional fatigue tests are conducted, and all specimens experience fracture at the spline on the minor-axis end. One of the fatigued and fractured specimens is shown in Figure 5. Upon macroscopic observation of the fracture surface of the main drive shaft (Figure 5a), plastic deformation of the material is evident at the fracture site. The crack origin is located near the tooth root of the spline, with the initial crack source situated in the transition zone adjacent to the tangency point between the arc and straight line at the bottom of the spline. The crack initiation occurs at the transition zone between the spline root and the tool withdrawal groove, which is the fatigue-critical region identified by our prior simulation analysis. As shown in Figure 5a, the crack initially propagates longitudinally along the tooth root under cyclic torsional loading, aligning with the direction of maximum shear stress. This initial stage is dominated by shear-dominated fatigue crack growth, consistent with the maximum shear stress criterion for torsional fatigue. Subsequently, as the crack extends and the local stress state evolves, the direction of propagation shifts to 45° relative to the longitudinal axis, which corresponds to the direction of the maximum principal stress. This shift arises from the redistribution of stress around the crack tip, where the opening-mode (Mode I) contribution becomes more significant. Macroscopic examination of the side surface of the spline reveals conchoidal striations indicative of cyclic loading (Figure 5b). Finally, during the final abrupt fracture stage, the crack propagates in a transverse direction across the shaft cross-section, driven by the rapid release of stored elastic energy and the resulting mixed-mode fracture. Fatigue cracks initiate on the surface of the specimen and then gradually propagate inward. From the overall view of the fracture surface, multiple crack initiation sites and regions of fatigue crack propagation from the surface inward can be identified, based on the macroscopic fracture morphology characteristics and crack propagation direction. Further magnification of the fatigue crack propagation region is shown in Figure 5b. Observations under low magnification indicate that fatigue fracture failure typically results from a single crack initiation source, with fatigue cracks predominantly originating from the surface or subsurface of the specimen. High-magnification examination of the crack region on the specimen surface reveals predominantly flat facets, where fatigue striations perpendicular to the crack propagation direction, as well as secondary cracks, can be clearly observed.

The results of the tests conducted under various equivalent strain amplitude conditions are presented in Table 2, where the experimental values for different specimens are separated by “/”.

As can be seen from Table 2, the torsional fatigue life of the drive shaft decreases with an increase in the equivalent strain amplitude. During monotonic torsional deformation failure, no macroscopic cracks are observed on the surface of the specimens; instead, failure occurs due to severe buckling deformation. However, in the case of torsional fatigue failure, obvious macroscopic cracks are visible on the surface of the specimens, indicating shear failure. Subjected to pure shear, the specimens experience maximum shear stress and shear strain distributed on their surfaces, causing cracks to initiate on the surface and propagate from the outside inward. The locations of the fatigue cracks are consistent with the results obtained from actual vehicles. The impact torque obtained from the bench test is greater than that from the real vehicle data. This discrepancy arises because, in the bench test, the busbar output gear of the transmission system is fixed to the base plate, approximating a rigid constraint. In contrast, for a real vehicle, on one hand, the vehicle possesses a certain amount of inertia; on the other hand, the components connected to the busbar output gear exhibit a degree of flexibility, which serves as a buffering mechanism.

**Figure 6:**
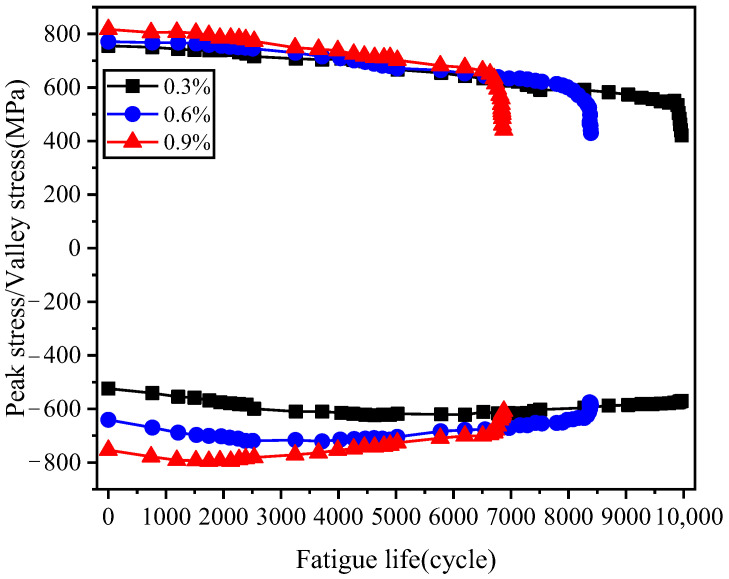
Peak/valley shear stress-life curve.

Figure 6 illustrates the peak/valley shear stress of the specimen under cyclic loading. During each group of fatigue tests, the sampling frequency of the extensometer is set to 5–10 times the fatigue loading frequency, which enables the capture of the peak and valley strain values for each cycle, and these are further converted to the corresponding peak and valley stress values. However, due to the extremely large volume of raw data, this study does not plot data from all cycles. Based on the research team’s experience, for each test group, 10 data points are selected for plotting from the initial 2500 cycles and from the interval of 3300 to 5000 cycles, respectively, with the time interval between consecutive data points decreasing gradually. From 5800 cycles until the end of the test, 15 sets of peak and valley stress values are recorded for each group, and the time interval between consecutive recordings also follows a “long-to-short” pattern. The peak/valley shear stress represents the maximum and minimum shear stresses experienced by the material during one cycle, characterizing its hardening/softening behavior and governing the material’s mean stress. Given the significant differences in fatigue life across various shear strain amplitudes, the shear stress response is depicted as a relationship between peak/valley stress and the cyclic fraction, where the *x*-axis employs the ratio of an arbitrarily given cycle number (*N*) to the cycle number at fatigue failure (*N_f_*). Overall, the peak/valley shear stress exhibits a gradual softening characteristic under six levels of strain amplitude, which aligns with the material’s cyclic damage behavior. As the shear strain amplitude increases, the peak shear stress initially exhibits an increasing trend during the initial stage. After approximately ten cycles, the peak shear stress values essentially converge and remain stable over an extended period, demonstrating that the maximum shear stress is insensitive to changes in strain amplitude. Regarding the valley shear stress, its initial value decreases significantly with an increase in the shear strain amplitude. For the same shear strain amplitude, the valley shear stress displays a hardening characteristic during the first few cycles, followed by a prolonged stable phase until crack initiation occurs. Under smaller shear strain amplitudes, the valley shear stress exhibits a continuous softening behavior and reaches a steady state in the later stages of cycling.

**Figure 7:**
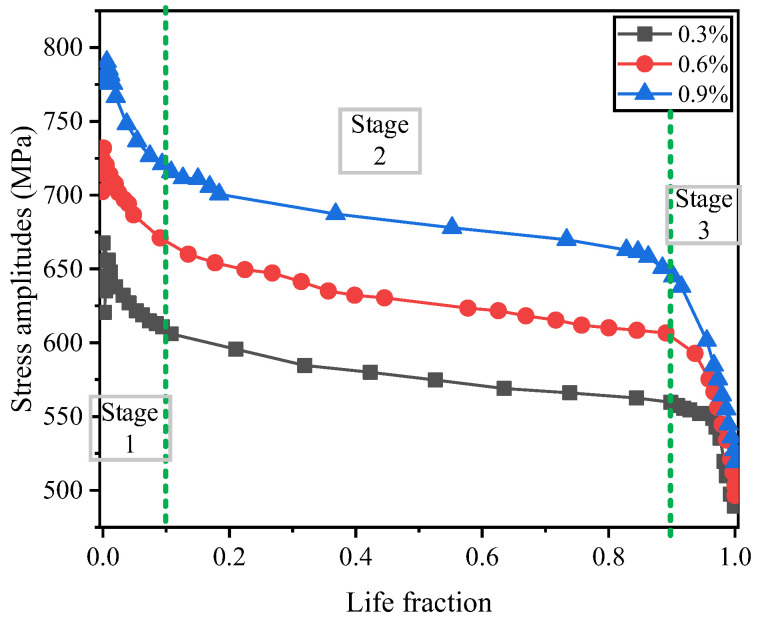
Shear stress amplitude-life fraction curve.

During the cyclic process, the variation in the material’s stress amplitude is governed by changes in the peak/valley stresses, and, to a certain extent, the variation in stress amplitude determines the material’s cyclic softening/hardening behavior. Figure 7 presents the stress amplitude evolution curves of the material under different strain amplitude controls. Life fraction represents the percentage of the current loading cycle number relative to the total number of cycles to failure. As the controlled strain amplitude increases, the stress amplitude exhibits distinct evolutionary behaviors. On the whole, the initial stress amplitude rises with an increase in the controlled strain amplitude, but the rate of increase gradually diminishes. For higher strain amplitudes (0.9%), the material softens rapidly, with essentially no hardening phase observed. In contrast, for lower strain amplitudes (0.3% and 0.6%), the drive shaft material demonstrates pronounced cyclic hardening characteristics during the early stages of fatigue. During the first 10% of the fatigue life cycles, cycles with larger strain amplitudes exhibit rapid softening, while those with smaller strain amplitudes demonstrate cyclic hardening. The second stage, accounting for approximately 10% to 90% of the fatigue life, is characterized by cyclic stability with gradual softening. The third stage, encompassing the final 10% of the cycles, also involves rapid stress softening, marked by a swift decline in stress amplitude and eventual failure.

**Figure 8:**
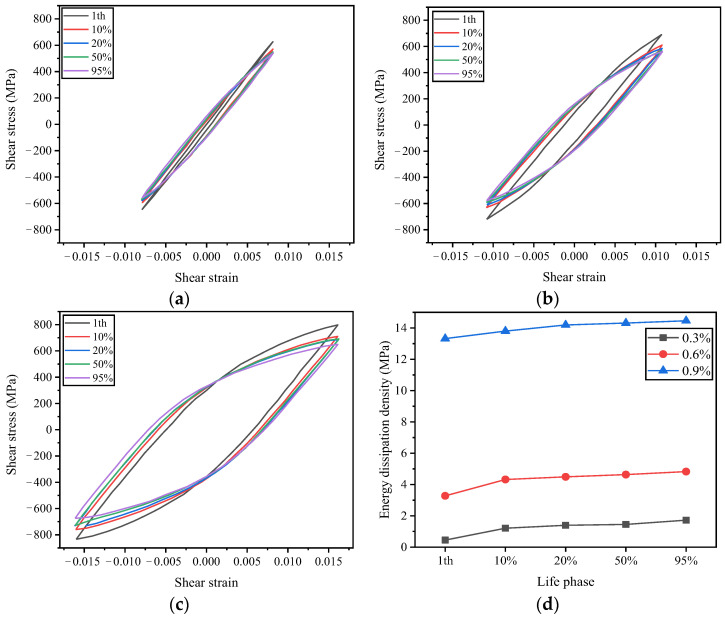
Stable cyclic shear stress-shear strain curve. (**a**) $\gamma_{a}=0.3\%$. (**b**) $\gamma_{a}=0.6\%$. (**c**) $\gamma_{a}=0.9\%$. (**d**) Variation in energy dissipation density.

**Figure 9:**
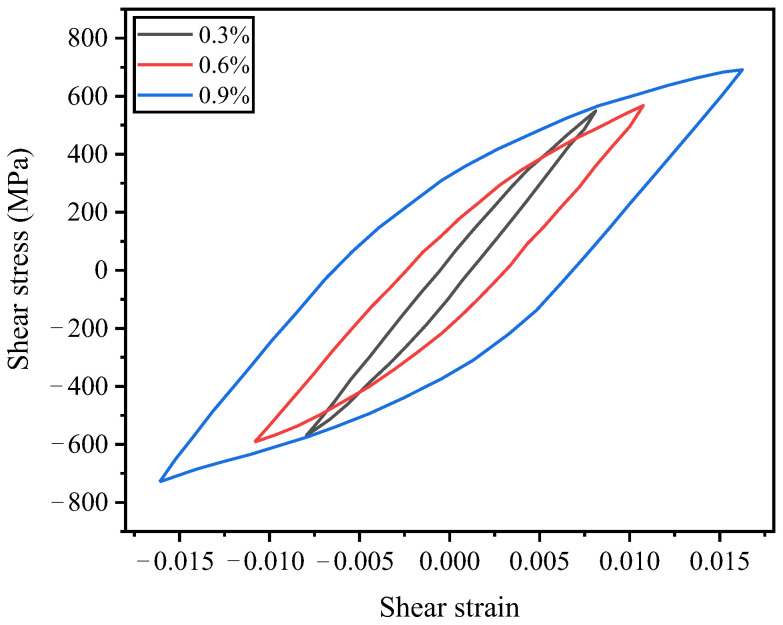
Stable cyclic shear stress-shear strain curve.

In this study, the hysteresis loop at half of the fatigue life is selected as the stable hysteresis loop to investigate the torsional fatigue characteristics. The data for the curves in Figure 8 and Figure 9 are derived from experimental measurements, with appropriate adjustments performed using Origin software in the same manner as described for Figure 4. This is a standard practice in experimental mechanics to improve the clarity of the stress–strain hysteresis loops, and the details are not repeated here for brevity. Figure 8a–c illustrates the evolution of the hysteresis curves for the drive shaft under three levels of equivalent strain amplitude as a function of the number of cycles. Due to the cyclic softening behavior of the material, the peak stress gradually decreases with an increasing number of cycles, while the width of the hysteresis curves widens—that is, the area of the hysteresis curves increases. This indicates that the energy dissipated during the cyclic process also rises, as shown in Figure 8d, implying a continuous accumulation of fatigue damage in the material due to the increasing plastic strain. As the softening trend becomes more gradual, the hysteresis curves also tend to stabilize.

Figure 9 presents the stress–strain hysteresis loops corresponding to the cyclic stability of 300M steel under different strain amplitudes. At a strain amplitude of 0.3%, the hysteresis loop area of the specimen is extremely narrow, indicating that plastic deformation is difficult to occur under this strain amplitude and that elastic deformation dominates the material’s deformation behavior. Such a stress–strain hysteresis loop is referred to as an elastic hysteresis curve. When the applied equivalent strain amplitude increases to 0.6%, the material undergoes relatively noticeable plastic deformation, and as the strain amplitude further increases, the plastic deformation of the material becomes increasingly pronounced. Notably, under different strain amplitudes, the shear stress-shear strain hysteresis loops for larger and smaller strain amplitudes exhibit varying degrees of crossover near the stress peak. This indicates that the degree and process of cyclic softening in the drive shaft are significantly dependent on the magnitude of the shear strain amplitude.

**Figure 10:**
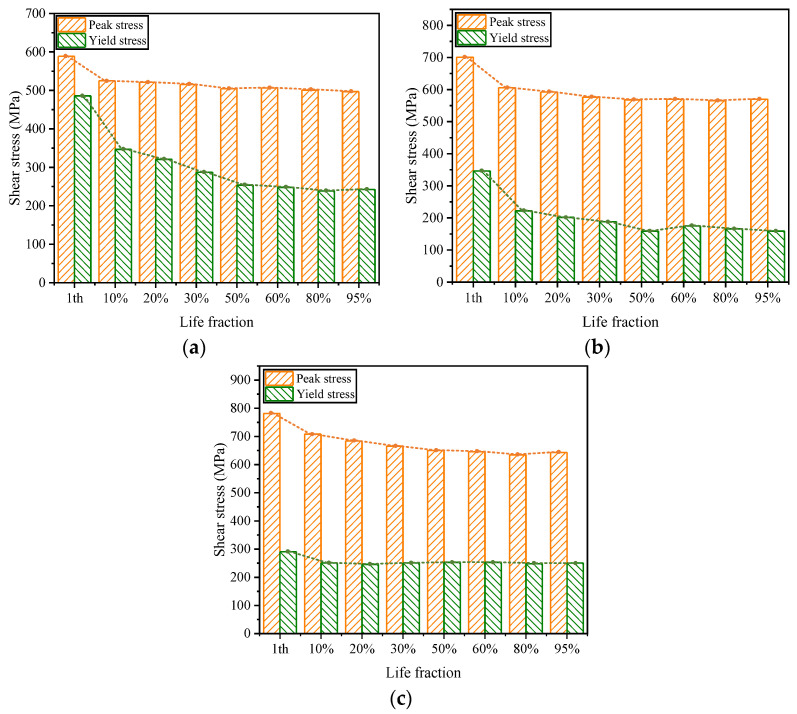
Peak stress and yield stress of hysteresis curve. (**a**) $\gamma_{a}=0.3\%$. (**b**) $\gamma_{a}=0.6\%$. (**c**) $\gamma_{a}=0.9\%$.

The elastic range of the hysteresis loops also varies at different cycle counts, meaning that the yield stress $\tau_{0.3}$ changes with the number of cycles. The variation in yield stress for hysteresis loops under different equivalent strain amplitudes as a function of the cycle count is illustrated in Figure 10, where the horizontal axis is represented by the cycle fraction $N/N_{f}$. In the figure, the red line indicates the changes in cyclic peak stress, while the blue line depicts the evolution of the yield stress $\tau_{0.3}$ with the number of cycles. Due to the influence of cyclic softening, the yield stress gradually decreases as the number of cycles increases, resulting in a reduction in the elastic range and an increase in plastic deformation. From the initial stage of cycling to around the cycle fraction $N/N_{f}=20\%$, the yield stress decreases at a relatively rapid rate. For large strain amplitudes, the evolution pattern of the yield stress is essentially consistent with the corresponding pattern of peak stress variation with the number of cycles. However, for a small strain amplitude of 0.3%, the change in yield stress during the initial stage of cycling is particularly pronounced, with a much faster rate of decrease compared to the corresponding decrease in peak stress. During the $N/N_{f}>25\%$ stage, the variation in yield stress tends to level off, and the changes in yield stress are generally in line with the corresponding changes in peak stress.

## 3. Finite Element Model for Drive Shaft Life Prediction

The drive shaft presents challenges due to its complex and bulky structure, diverse service conditions, and intricate material constitutive calculations. The analysis of its damage evolution involves solving highly nonlinear problems, making it difficult to construct a full-scale finite element model of the drive shaft that meets the requirements for multi-scale damage evolution analysis. In response to the aforementioned issues, the research team conducts an analysis of the influence of operating conditions and structural dimensions on the mechanical response of the drive shaft [23,24,25]. This study further develops a hierarchical material constitutive model for the life-vulnerable zone of the drive shaft, which not only enhances the accuracy of mechanical response analysis but also reduces the computational cost associated with highly nonlinear finite element simulations of complex models.

**Figure 11:**
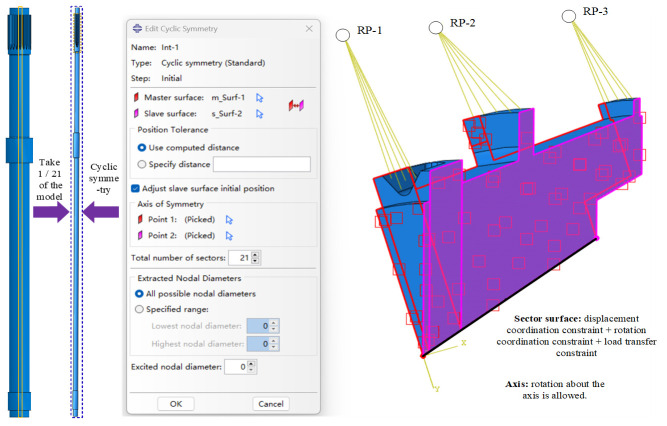
Drive shaft model.

Based on experimental results and real-vehicle observations, it is evident that cracks consistently occur at the spline relief groove on the short-shaft end. To save computation time, the intermediate spline and the spline on the long-shaft end are simplified as smooth shafts. The drive shaft model, as shown in Figure 11, is constructed using ABAQUS 2021 software. The modulus of the spline on the short-shaft end is 21. Since the majority of the overall drive shaft model remains in the elastic deformation stage, an elastoplastic constitutive model is selected. Given that the drive shaft exhibits an axisymmetric model at this time, taking one twenty-first of the model and setting up the cyclic symmetric interaction provided by the ABAQUS software can further reduce computational costs. The cyclic symmetry boundary condition adopted in Abaqus is centered on enforcing the displacement and rotation coordination between a pair of circumferential sections, which enables the establishment of a single-sector finite element model and drastically reduces the computational cost. This boundary strategy is particularly applicable to rotationally symmetric structural components including impellers, splines, disks, and annular parts. For the cyclic symmetry modeling, all nodes located on the rotational symmetry axis are constrained in terms of radial and circumferential displacements, while only the rotational degree of freedom around the axis is permitted to maintain the geometric invariance of the model during rotational loading. Subsequently, the two circumferential sections of the single-sector model are defined as the master surface and slave surface, respectively, and three mandatory constraint requirements are imposed: displacement coordination, which means the displacements of nodes on the slave surface are equivalent to those of the corresponding nodes on the master surface after rotating by the sector angle around the symmetry axis; rotation coordination, which ensures no separation, dislocation or warpage occurs at the paired circumferential sections; load transfer, which allows circumferential loads such as torque and centrifugal force to be uniformly transmitted to the entire annular structure through this constraint. Torque is applied to the spline on the short-shaft end, while the spline on the long-shaft end is fixed (with displacement constraints on the tooth surfaces). This represents an extreme operating condition and also the most hazardous limit condition for the short-shaft end under stress.

Most regions of the drive shaft are in the elastic deformation stage. Combining the data from monotonic torsion tests, the relationship between shear stress τ and shear strain γ is commonly expressed using the Ramberg–Osgood equation [23]:

(1) $$\gamma =\gamma_{e}+\gamma_{p}=\frac{\tau }{G}+{\left(\frac{\tau }{K_{0}}\right)}^{\frac{1}{n_{0}}}$$

where $\gamma_{e}$ represents the elastic shear strain, $\gamma_{p}$ denotes the plastic shear strain, $K_{0}$ is the shear strength coefficient, and $n_{0}$ stands for the shear strength exponent.

The fundamental mechanical parameters of the drive shaft under monotonic torsion at room temperature are obtained by fitting the shear stress–shear strain curve of monotonic torsion derived from the experiments, as shown in Table 3.

**Figure 12:**
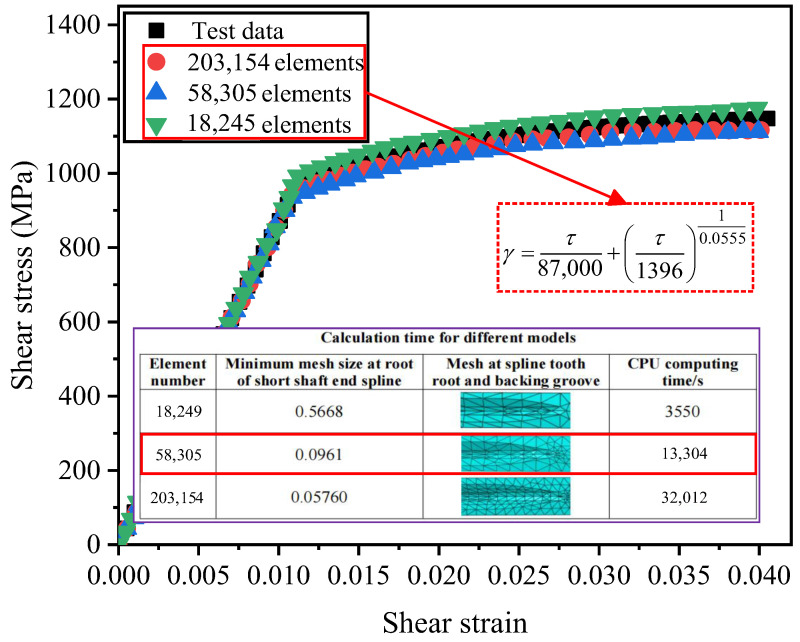
Stress variation curves corresponding to different models.

The material properties of the overall model of the drive shaft are set according to Equation (1), and then a convergence analysis is conducted to determine an appropriate mesh size for the element model. Tetrahedral elements are selected to enhance mesh generation efficiency, with the element type being C3D10. The right spline section is the key area of concern, and the mesh in this spline section is refined. The time histories of simulation duration and the maximum stress point corresponding to models with different mesh counts are illustrated in Figure 12. The analysis revealed that when the number of mesh elements exceeded 58,305, the differences in stress results are minimal, and the computational time remained within an acceptable range. Therefore, the model is discretized into 58,305 mesh elements, with the minimum mesh size at the root of the spline teeth on the short-shaft end being 0.0961. Given that the simulation results are based on the shear stress–strain relationship expression, a comparison with experimental results demonstrated that Equation (1) exhibited high curve-fitting accuracy.

**Figure 13:**
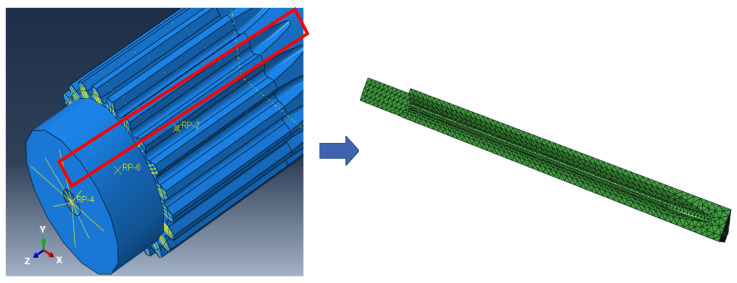
The sub-model of the drive shaft.

Based on the team’s previous research findings [23,24,25], it is known that cracks always initiate at the relief groove of the spline on the short-shaft end. To further economize on computational time, the sub-modeling technique inherent in ABAQUS is utilized to construct a more finely meshed model of the life-vulnerable zone within the plastic region encompassing the maximum stress point, as depicted in Figure 13. This sub-model is excised from the macroscopic model, ensuring alignment within the identical coordinate system, and its boundary conditions are defined by the displacement deformations observed in the macroscopic model.

The life-vulnerable zone model exhibits various mechanical phenomena under cyclic loading, including cyclic hardening or softening, ratcheting effect, and Bauschinger effect. Therefore, a sophisticated constitutive model is required to accurately characterize the elastoplastic cyclic response behavior of the material. The complex cyclic deformation behavior, coupled with the combined effects of cyclic hardening and softening, poses a significant challenge to the mathematical description of the elastoplastic response in numerical simulation studies. In this work, our research team takes the cyclically stable hysteresis loop as the foundation for macroscopic constitutive modeling [19]. Based on the Von Mises isotropic yield criterion, a mixed-hardening cyclic plastic constitutive model considering the Bauschinger effect of the material is established by combining the superimposed Voce isotropic hardening model and the A-F nonlinear kinematic hardening model [19]. This model is developed on the basis of the Chaboche model, and the yield function f can be expressed as follows [23]:

(2) $$f(\sigma -\alpha ,\sigma_{F})=\sqrt{\frac{3}{2}(s-a):(s-a)}-\sigma_{F}=0$$

where α denotes the back stress tensor, which represents the center of the yield surface. $\sigma_{F}$ refers to the frictional stress, which stands for the radius of the yield surface. s is the deviatoric stress tensor, a denotes the deviatoric back stress tensor, and σ represents the total stress tensor.

According to the generalized orthogonality assumption, the direction of the plastic flow increment is consistent with the normal direction of the yield surface, and the plastic strain increment $d\;\varepsilon^{p}$ can be expressed as follows [23]:

(3) $$d\;\varepsilon^{p}=d\lambda \cdot \frac{\partial f}{\partial \sigma }$$

where $N=\frac{\partial f}{\partial \sigma }$ denotes the normal direction of the yield surface, and dλ represents the time-independent plastic multiplier.

Generally, the mixed hardening rule is adopted to characterize the kinematic hardening and isotropic hardening of the material. For the kinematic hardening component, the classical A-F nonlinear kinematic hardening model, which is capable of describing the Bauschinger effect and transient behavior of materials, is utilized. Furthermore, the superimposed Voce nonlinear isotropic hardening criterion is employed to express the isotropic stress, and the final material flow stress can be expressed as follows [23]:

(4) $$\sigma =\sigma_{0}+Q_{1}[1-\exp(-2Nb_{1}\varepsilon^{p})]+Q_{2}[1-\exp(-2Nb_{2}\varepsilon^{p})]+\frac{2}{3}\sum_{i=1}^{M}\frac{C_{i}}{r_{i}}[1-\exp(-r_{i}\varepsilon^{p})]$$

where Q and b denote the maximum variation in the yield surface radius and the variation rate of the yield surface size with plastic strain, respectively. M represents the back stress series, and $C_{i}$ and $r_{i}$ are the kinematic hardening material parameters.

**Figure 14:**
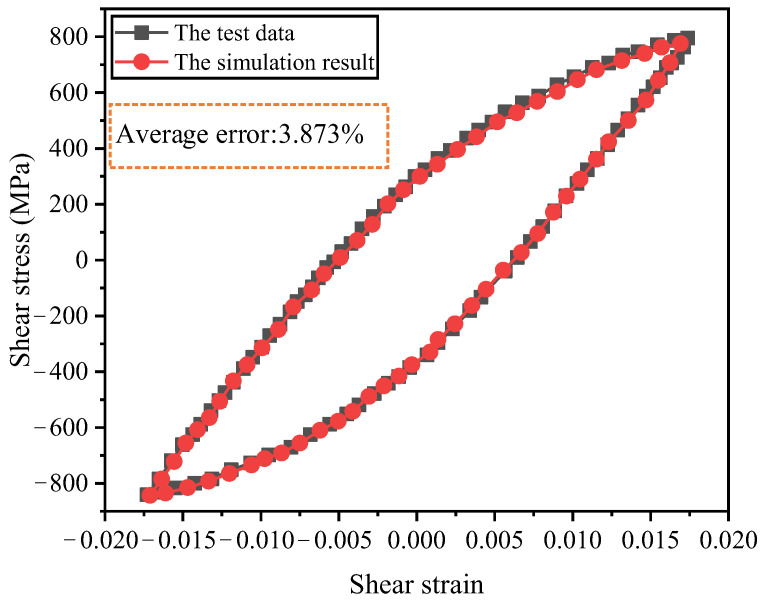
The simulation and test results of the stable hysteresis loop.

The mixed-hardening cyclic plastic constitutive model involves a considerable number of parameters, with an extremely sophisticated nonlinear functional correlation existing between the model parameters and the stress–strain response. In this study, our research team implemented finite element parameter inversion of the constitutive model via the combination of the Kriging surrogate model and genetic algorithm [23]. Given that this method demands repeated invocation of the finite element model for simulation calculations, a drive shaft sub-model is utilized to fit the stable hysteresis curve at 0.9% strain amplitude, so as to reduce the computational cost and realize the precise and efficient identification of the constitutive model parameters. The obtained parameters of the constitutive model are listed in Table 4. To verify the accuracy of the constitutive model, the stress–strain results were extracted from the smooth cylindrical section of the short shaft end adjacent to the end spline. As illustrated in Figure 14, a comparison between the simulated results and experimentally measured data for the 0.9% stable hysteresis loop demonstrates that the improved constitutive model achieves an average computational error of 3.873%, accurately capturing the cyclic mechanical behavior of 300M steel.

## 4. Low Cycle Fatigue Life Prediction

At present, research on fatigue life prediction methods for mechanical structures has developed a corresponding theoretical framework, yet numerous pressing issues still remain to be resolved. This study focuses on the impacts of mean stress, load interaction, and strength degradation on fatigue life, and proposes improvements to fatigue life prediction methods from two aspects: low-cycle fatigue life prediction models and fatigue cumulative damage theory.

### 4.1. Improved Low Cycle Fatigue Life Prediction Model

In engineering applications, high-strength steels are prone to stress concentration at defects under the action of relatively high cyclic stresses, leading to localized plastic deformation and, consequently, the initiation of low-cycle fatigue. The strain-fatigue life curve method is commonly employed for fatigue life prediction and assessment under low-cycle fatigue conditions, where the cyclic response of the material is described by both elastic stress–strain and plastic stress–strain behaviors. Manson and Coffin proposed establishing a relationship between total strain and life, with the strain-fatigue formula expressed as follows [24]:

(5) $$\frac{\Delta \gamma }{2}=\frac{\Delta \gamma_{e}}{2}+\frac{\Delta \gamma_{p}}{2}=\frac{{\tau^{\prime }}_{f}}{G}{\left(2N_{f}\right)}^{b}+{\gamma^{\prime }}_{f}{\left(2{N_{f}}^{\prime }\right)}^{c}$$

where ${\tau^{\prime }}_{f}$ represents the fatigue strength coefficient, b denotes the fatigue strength exponent, ${\gamma^{\prime }}_{f}$ stands for the fatigue plasticity coefficient, and c indicates the fatigue plasticity exponent, ${N_{f}}^{\prime }$ is the predicted fatigue life.

The Manson–Coffin equation is derived under the condition of symmetric cyclic loading, i.e., when the stress ratio *R* = −1. In engineering practice, however, asymmetric cyclic loading is almost ubiquitous, necessitating an average stress correction. In this study, referencing the average stress correction method proposed by Wang et al. [10], a low-cycle fatigue prediction model accounting for the average shear stress $\tau_{m}$ effect is established as follows [24]:

(6) $$\tau_{\max}^{1-\omega }\tau_{a}^{\omega }\gamma_{a}=\frac{{\left({\tau^{\prime }}_{f}\right)}^{2}}{G}{\left(2{N_{f}}^{\prime }\right)}^{2b}+{\tau^{\prime }}_{f}\gamma \prime_{f}{\left(\frac{{\tau^{\prime }}_{f}-\tau_{m}}{{\tau^{\prime }}_{f}}\right)}^{c/b}{\left(2{N_{f}}^{\prime }\right)}^{b+c}$$

where $\gamma_{a}$ represents the shear strain amplitude, and $\tau_{a}$ denotes the shear stress amplitude, ω is a material constant that can be used to describe the sensitivity degree of different materials to the influence of mean stress. A larger value of ω indicates a lower sensitivity of the material to the influence of mean stress.

Dowling [26] investigated the relationship between the material constant ω and the fatigue strength $\tau_{u}$ of steel and aluminum alloys, pointing out that as a increases, there is a tendency for the fatigue strength to decrease. Wang et al. [10] obtained a mathematical expression for 300M steel through linear fitting of test data:

(7) $$\omega =-0.00026\tau_{u}+0.75$$

However, during the fatigue failure process of materials, with the continuous application of fatigue loads, the fatigue damage of components gradually accumulates, and mechanical property indicators such as fatigue strength, stiffness, and toughness also gradually decline. Considering that the degradation rate of fatigue strength is relatively slow in the initial stage of fatigue loading and rapidly deteriorates at the final stage of fatigue loading, this study establishes a macroscopic phenomenological fatigue strength degradation model. Combining the improvements made by Li et al. [19] to the M-H model, the fatigue strength under n-cycle loading R(n) is as follows:

(8) $$R(n)=A-B\exp(e^{n/{N_{f}}^{\prime }}-1)$$

In addition, R(n) must also meet the following conditions: at the initial loading stage, the component strength does not decrease, with its initial value being the material’s initial shear fatigue strength limit $\tau_{u}$. Moreover, at the end of the loading process, when the load cycles reach the predicted fatigue life ${N_{f}}^{\prime }$, the residual strength equals the applied load level $\tau_{i}$. The values of A and B are determined as follows:

(9) $$\left\{\begin{array}{c} A=\frac{\exp(e-1)\tau_{u}-\tau_{i}}{\exp(e-1)-1}\\ B=\frac{\tau_{i}-\tau_{u}}{\exp(e-1)-1} \end{array}\right.$$

At this point, the expression for the residual fatigue strength R(n) is as follows:

(10) $$R(n)=\tau_{u}+\frac{\left(\tau_{u}-\tau_{i}\right)e^{n/N_{f}}}{\exp\left(e-1\right)-1}$$

The expression for the material constant ω is transformed into the following form:

(11) ω=−0.00026R(n)+0.75

By combining Equation (6) and Equation (10), the final low-cycle fatigue prediction model considering the mean stress effect is established as follows:

(12) $$\begin{array}{l} \tau_{\max}^{0.25+0.00026(\tau_{u}+\frac{\left(\tau_{u}-\tau_{i}\right)e^{n/{N_{f}}^{\prime }}}{\exp\left(e-1\right)-1})}\tau_{a}^{0.75-0.00026(\tau_{u}+\frac{\left(\tau_{u}-\tau_{i}\right)e^{n/{N_{f}}^{\prime }}}{\exp\left(e-1\right)-1})}\gamma_{a}=\frac{{\left({\tau^{\prime }}_{f}\right)}^{2}}{G}{\left(2{N_{f}}^{\prime }\right)}^{2b}\\ +{\tau^{\prime }}_{f}{\gamma^{\prime }}_{f}{\left(\frac{{\tau^{\prime }}_{f}-\tau_{m}}{{\tau^{\prime }}_{f}}\right)}^{c/b}{\left(2{N_{f}}^{\prime }\right)}^{b+c} \end{array}$$

The fatigue parameters to be determined by fitting in Equation (12) are the fatigue strength coefficient, fatigue strength exponent, fatigue ductility coefficient, and fatigue ductility exponent, respectively. The shear modulus *G* is obtained from material handbooks, and all other values are either experimentally set or measured. The fatigue parameters obtained by fitting the experimental data are listed in Table 5.

**Figure 15:**
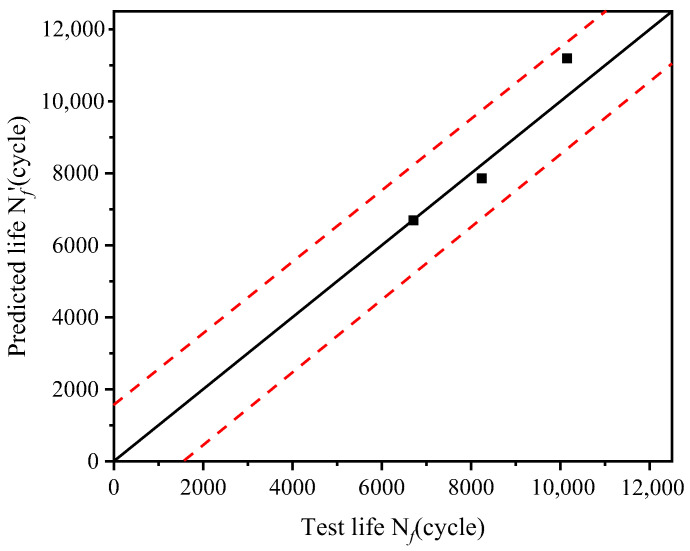
Comparison results between predicted and measured torsional fatigue life values.

The fatigue parameters are obtained by fitting the test data, as shown in Table 3. After determining the parameter values in Equation (12), the validity of its description of the torsional fatigue behavior of the drive shaft could be evaluated. The effectiveness of the low-cycle fatigue prediction model considering the mean stress effect (Equation (12)) in describing the torsional fatigue behavior of the drive shaft was verified using three sets of test data. The validation results are presented in Figure 15, where the *x*-axis represents the measured values (test fatigue life $N_{f}$), the *y*-axis represents the predicted values (predicted fatigue life ${N_{f}}^{\prime }$), the central solid black line denotes the ideal prediction with zero error, and the region between the two red dashed lines represents the two-fold error band. The predicted fatigue life of Equation (12) all fall within the two-fold error band, demonstrating that the proposed model can well describe the torsional fatigue life behavior of the drive shaft.

### 4.2. Improved Fatigue Cumulative Damage Theory

The M-H model, which takes into account the influence of loading sequence on cumulative damage, represents a relatively typical nonlinear fatigue cumulative damage model. Based on cumulative damage criteria, it derives the failure criterion for components under multilevel loading conditions. The fatigue cumulative damage criterion under multilevel loading is as follows [25]:

(13) $$\left\{\begin{array}{c} D_{e,i}={\left({\left({\left({\left(\frac{n_{1}}{N_{f_{1}}}\right)}^{\alpha_{1,2}}+\frac{n_{2}}{N_{f_{2}}}\right)}^{\alpha_{2,3}}+\frac{n_{3}}{N_{f_{3}}}\right)}^{\alpha_{3,4}}+\ldots +\frac{n_{i-1}}{N_{f_{i-1}}}\right)}^{\alpha_{i-1,i}}+\frac{n_{i}}{N_{f_{i}}}=1\\ \alpha_{i-1,i}={\left(\frac{N_{f_{i-1}}}{N_{f_{i}}}\right)}^{0.4} \end{array}\right.$$

The M-H model delineates a nonlinear relationship between cyclic damage and fatigue life, yet it falls short in considering the effects of load interaction. Generally, under a H-L (High-to-Low) loading sequence, it is postulated that the preceding higher load level will accelerate the cumulative damage process of the subsequent lower load level, leading to an increase in damage induced by the current stress level. Conversely, under an L-H (Low-to-High) loading sequence, the preceding lower load level is believed to induce a crack retardation effect, thereby retarding the cumulative damage process of the subsequent higher stress level and resulting in a decrease in damage generated by this stress level. This indicates that a portion of the work performed during the stress cycle of the subsequent load level will be expended in overcoming other influencing factors, with a consequent reduction in the work that truly contributes to structural damage. Typically, the influence of load interaction on fatigue cumulative damage can be captured by introducing the stress ratio between consecutive load levels. Drawing on Zhao et al.’s [20] modification of the M-H model, this study introduces a load ratio $k_{i}$ modified life ratio characteristic to reflect the impact of H-L and L-H loading sequences on cumulative fatigue.

(14) $$k_{i}=\max\left(\frac{\tau_{i-1}}{\tau_{i}},\frac{\tau_{i}}{\tau_{i-1}}\right)$$

In fact, the fatigue cumulative damage of metal components represents a complex issue influenced by the interplay of multiple parameters, exhibiting dynamic, nonlinear, and fuzzy (yet irreversible) variations. In this study, the extent of the influence exerted by load interaction on the damage process is quantitatively described using a membership function, thereby further refining the nonlinear cumulative damage model. Based on literature review and fatigue test results [20], the membership function reflecting the impact of adjacent loads on fatigue damage is represented by a lower-bound truncated normal distribution function:

(15) $$f(\tau_{i})=\left\{\begin{array}{cc} 1-e^{-{\left(\frac{\tau_{i}-0.85R(n)}{0.22R(n)}\right)}^{2}}, & \tau_{i}\geq 0.85R(n)\\ 0 & \tau_{i}<0.85R(n) \end{array}\right.$$

The variable R(n) represents the residual fatigue strength, whose degradation is intricately linked to the accumulation of damage and can comprehensively reflect the extent of damage sustained. R(n) is evaluated for the specific stress level by following the procedure detailed previously in Equations (8)–(10). Therefore, it is essential to incorporate the influence of fatigue strength degradation into the fatigue cumulative damage model for accurate fatigue life prediction.

Ultimately, by integrating Equation (13) through Equation (15), a nonlinear fatigue cumulative damage model that comprehensively accounts for load interaction and fatigue strength degradation is established as follows:

(16) $$\left\{\begin{array}{c} D_{e,i}={\left({\left({\left({\left(\frac{n_{1}}{N_{f_{1}}}\right)}^{\alpha_{1,2}}+\frac{n_{2}}{N_{f_{2}}}\right)}^{\alpha_{2,3}}+\frac{n_{3}}{N_{f_{3}}}\right)}^{\alpha_{3,4}}+\ldots +\frac{n_{i-1}}{N_{f_{i-1}}}\right)}^{\alpha_{i-1,i}}+\frac{n_{i}}{N_{f_{i}}}=1\\ \alpha_{i-1,i}={\left(\frac{N_{f_{i-1}}}{N_{f_{i}}}\right)}^{{0.4k_{i}}^{f\left(\tau_{i}\right)}} \end{array}\right.$$

## 5. Results

Regarding the drive shaft, restricted by the substantial time and economic costs required for its fatigue tests, this study adopted finite element analysis instead of experimental tests to acquire the stress and strain data of the drive shaft. Subsequently, the low-cycle fatigue life prediction model proposed in Equation (12) was utilized to predict the single-stage load fatigue life of the drive shaft, and Equation (16) was further adopted to calculate its cumulative fatigue under multi-stage loading.

### 5.1. Model Validation

Taking the experimental data of three materials as examples [10], the correctness and effectiveness of the proposed improved model are verified by comparing experimental results with predicted results. The stress, strain, and other relevant data obtained from the fatigue tests of specimens reported in References [10,19] are substituted into the proposed fatigue life prediction model (Equation (12)) to generate the corresponding life prediction results. The three materials are GH4133, carbon steel, and 7075-T651. Meanwhile, to validate the rationality and effectiveness of the improved model, a comparative analysis is conducted with the prediction results of the M-C model and the SWT model. The comparative analysis results of the prediction accuracy between the improved model and traditional models are shown in Table 6. The prediction error is defined as the percentage of the absolute difference between the predicted value and the experimental value relative to the experimental value. It can be observed that all models are capable of achieving a favorable prediction error within 50%. Among them, the prediction error of the SWT model is within 25%, while the prediction accuracy of the improved model is significantly superior to that of traditional models, with a prediction error not exceeding 15%. Therefore, it is proven that the improved model proposed in this study is correct and effective.

Since the fatigue test loading specified in the relevant standard [27] only extends up to three levels and follows a low-to-high loading sequence, this study takes two-level high-low and low-high loading, as well as three-level low-high loading, as examples. Fatigue test values for two-level loading on C35 material specimens and three-level loading on Q235 material specimens are selected [20].

The stress, strain and other relevant data acquired from the specimen fatigue tests in Reference [20] are substituted into the proposed fatigue life prediction model (Equation (12)) to obtain the single-stage load fatigue life prediction results, and these single-stage life results are further substituted into Equation (16) to calculate the fatigue cumulative damage under multi-stage loading. As the experimental data only provide single-stage damage degree values for the specimens, to facilitate direct comparison with the experimental values and following established research practices in this field, the predicted values of the highest-level damage degree, along with the experimental values and relative prediction errors, are compiled in Table 7.

The highest-level damage degree generally refers to the damage value induced by the last (highest stress) load level in a multi-level loading sequence, which represents the damage portion assigned to the last level rather than the total damage of the material. Compared to the predicted values of the Palmgren-Miner model and the traditional M-H model, the predicted values of the improved model are closer to the experimental values. The fatigue damage ratios predicted by the improved model all fall within the allowable prediction error range of 25%, and most of them meet the favorable prediction error range of 20%. For prediction errors under different loading modes, the average error of the improved model is significantly reduced.

### 5.2. Drive Shaft Life Prediction Results

**Figure 16:**
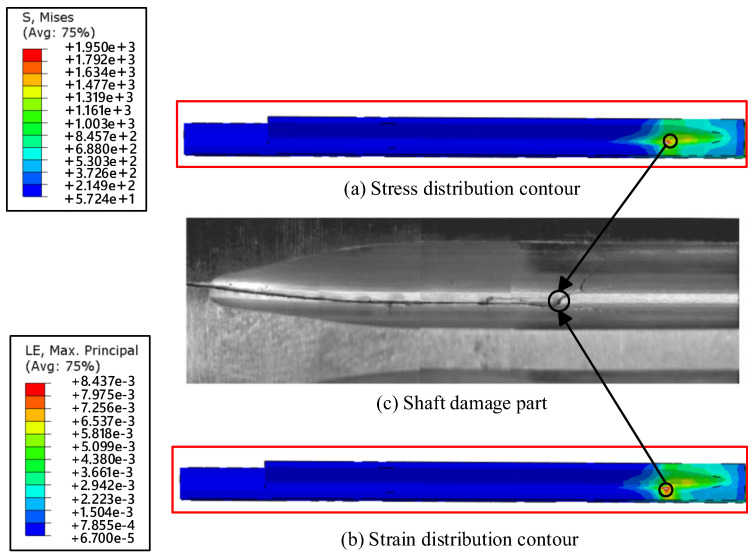
Stress and strain distribution cloud map.

A torque of 15,909 Nm is applied to the spline at the short-shaft end, while the spline at the long-shaft end is fixed (with tooth surface displacement constraints). This represents an extreme operating condition and also the most critical limit condition where the short-shaft end experiences the highest stress. By incorporating the boundary constraints and loads, a finite element analysis is performed on the drive shaft model, yielding results in the form of stress distribution contour plots and deformation contour plots, as shown in Figure 16a,b. The maximum von Mises stress value is 1950 MPa, which exceeds the ultimate strength (1860 MPa).

The maximum stress occurs at the junction between the relief groove and the spline on the short-shaft end, and the average stress on the short shaft is significantly higher than that on the long shaft. Due to factors such as geometric discontinuities and inhomogeneous microstructure within the drive shaft itself, stress concentration can occur locally, leading to localized yielding at the notch root and the formation of a plastic zone. Under short-term impact loading, slight plastic deformation takes place, accompanied by the emergence of microcracks. The actual crack initiation region obtained from the bench test is shown in Figure 16c. The strain damage caused by low-cycle fatigue results in crack formation at the junction between the relief groove and the spline.

The finite element simulation analysis results of the drive shaft sub-model are shown in Figure 16. The maximum Mises stress is 1950 MPa, and the maximum equivalent strain is 0.008437, with an elastic strain of 0.007332 and a plastic strain of 0.001105. Based on the finite element simulation results of the drive shaft, substituting the values into Equation (12) yields a low-cycle fatigue cycle count of 16,126 for the drive shaft, indicating that the drive shaft can withstand 16,126 torque cycles. According to the principle of equal damage, a fatigue test is conducted on the drive shaft until fatigue cracks appear.

**Figure 17:**
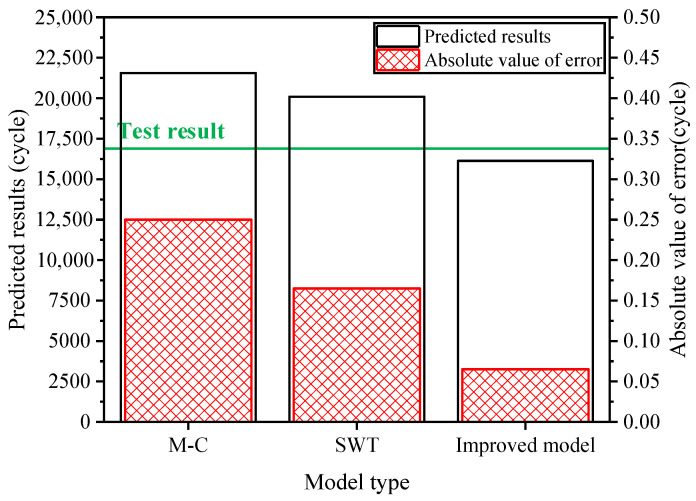
Life prediction results.

Combining the low-cycle fatigue life prediction results, the test is divided into multiple stages. In the initial test stage, 6000 start-up conditions are simulated, and the integrated transmission system is disassembled to inspect the damage to the drive shaft. In both the second and third test stages, 3000 start-up conditions are simulated, followed by damage inspections. In the fourth to sixth stages, 1500 start-up conditions are simulated each time, and no cracks are detected through flaw detection. In the seventh stage, 750 start-up conditions are simulated. The results indicated that after enduring 17,250 start-up conditions, significant fatigue cracks were observed at the tooth root of the spline on the minor-axis end of the drive shaft. The location of these fatigue cracks is consistent with the results obtained from actual vehicle testing. Considering that the final fracture life of the drive shaft is essentially the same as its crack initiation life, the experimentally tested drive shaft underwent fatigue fracture after experiencing 17,250 impact torque cycles, with a prediction error margin not exceeding ±10%. This validates the feasibility of the fatigue life prediction method for the main shaft. As illustrated in Figure 17, compared to traditional fatigue prediction models, the improved model yields life predictions that are closer to the experimental values, demonstrating its significant superiority.

**Figure 18:**
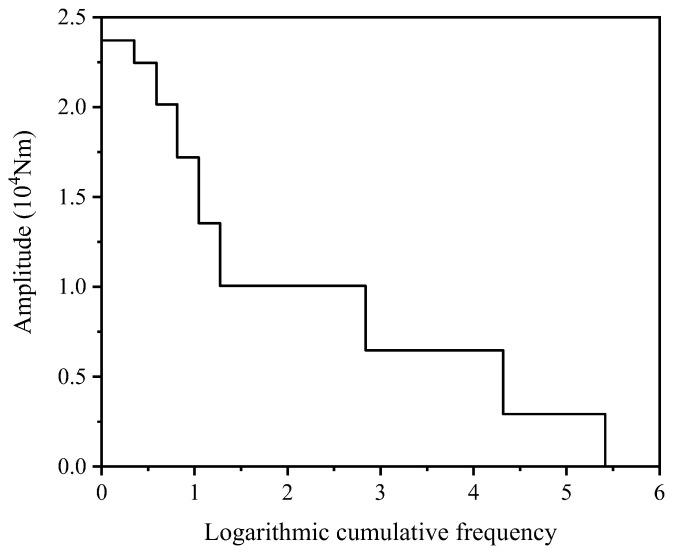
Torque load spectrum of the drive shaft.

**Figure 19:**
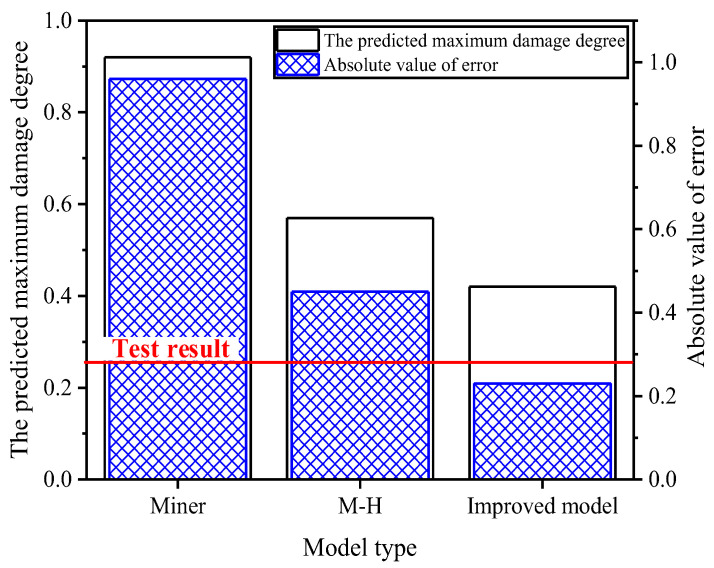
Life prediction results.

During its service life, the drive shaft is subjected to various operating condition loads. By comprehensively applying mathematical statistics and signal analysis, combined with the rainflow counting method, the torque load spectrum of the drive shaft is compiled, as shown in Figure 18, with the highest-level damage degree in the test being 0.263. Using the finite element analysis model of the drive shaft, fatigue damage hotspots are identified. By leveraging an improved low-cycle fatigue life prediction model and an enhanced fatigue cumulative damage criterion, the highest-level damage degree at the maximum stress location of the drive shaft is estimated to be 0.42, with a prediction error not exceeding ±25%, as depicted in Figure 19. Compared to the traditional Miner’s rule and M-H criterion, the improved fatigue cumulative damage criterion exhibits significantly higher prediction accuracy.

## 6. Discussion

In this study, a fatigue life prediction model considering the mean stress effect and fatigue strength degradation is established by introducing dynamically degraded fatigue strength into the mean stress-corrected SWT model. Meanwhile, a fatigue cumulative damage model accounting for load interaction and fatigue strength degradation is developed. Finally, a fatigue life prediction method for transmission shafts considering material cyclic characteristics is proposed. The results demonstrate that the proposed life prediction method can effectively improve the fatigue life prediction accuracy of transmission shafts.

A notable advantage of the proposed modeling approach lies in its general applicability to various engineering metal materials beyond the high-strength alloy steel investigated in this study. The core mechanism of the model—describing fatigue damage evolution by linking mean stress sensitivity, cyclic plastic deformation, and dynamic strength degradation—is physically consistent with the fatigue failure nature of most structural metals. For materials exhibiting different cyclic behaviors (e.g., cyclic hardening materials, lightweight alloys with low ductility, or non-ferrous metals with distinct mean stress sensitivity), the model can be adapted by calibrating key parameters related to cyclic deformation characteristics and strength degradation rates. Therefore, this modeling strategy is not limited to the tested steel but can be extended to a wide range of engineering materials with different cyclic mechanical behaviors. The proposed method can be applied to similar critical components in aerospace, rail transit, automotive manufacturing, and other engineering fields, especially for key parts subjected to complex variable loads and extreme service environments. The research outcomes provide a reliable theoretical basis for fatigue performance evaluation and life design, which is of great engineering value for enhancing the service safety, operational reliability, and economic efficiency of mechanical equipment.

However, this study still has certain limitations, and many factors affecting the accuracy of life prediction need further discussion. For example, how to quantitatively describe the correlation between the material’s sensitivity to mean stress and its cyclic characteristics, and how to reflect the relationship between the material’s fatigue strength degradation mechanism and the cumulative mechanism of cyclic plastic deformation. Due to the limitations of computational cost and time, this study does not further verify the rationality of the drive shaft submodel. These issues also point out the direction for the team’s future research, and the team will further improve the prediction capability of the life prediction method targeting these limitations.

In addition, considering that the fatigue-critical region of the drive shaft is dominated by shear stress under pure torsional loading, with only a minor contribution from additional stresses, the torsional fatigue behavior of the drive shaft was initially simplified as a uniaxial fatigue problem in this study, and the effect of component thickness was neglected. The drive shaft is indeed subjected to a multiaxial stress state under torsional loading. Moreover, due to its relatively large thickness, the through-thickness stress gradient and constraint effect play a non-negligible and important role in fatigue behavior [15,16,17]. Consequently, even the sole use of a multiaxial fatigue model is insufficient to fully characterize the damage evolution process. It is well known that fatigue is governed by numerous complex factors, and there always exists a discrepancy between idealized simplifications and real engineering problems. Scholars in the fatigue community have been continuously striving to narrow this gap so as to improve the consistency between fatigue damage predictions and actual service conditions. Therefore, to achieve a more accurate fatigue life prediction for the drive shaft, our research team will conduct an in-depth investigation on multiaxial fatigue life prediction methods considering the thickness effect in future work.

## 7. Conclusions

This study proposes a low-cycle fatigue life prediction method for drive shafts that accounts for load effects and strength degradation. Mechanical properties of the drive shaft are obtained through experimental testing. Based on the experimental data, a finite element model of the drive shaft that considers material cyclic characteristics is established. A fatigue life prediction method that incorporates load effects and fatigue strength degradation is then proposed. Finite element analysis is conducted to obtain the stress–strain response at critical locations of the drive shaft. The fatigue life of the drive shaft under a load spectrum is calculated by combining this response with the improved fatigue life prediction method. The conclusions are as follows:

1. The material of the drive shaft exhibits significant cyclic softening characteristics, with the degree and process of cyclic softening being notably dependent on the magnitude of the shear strain amplitude. For small strain amplitudes, the size of the hysteresis loop changes more significantly with an increase in the number of cycles. Under the same shear strain amplitude, the elastic range of the hysteresis loop for the drive shaft decreases as the number of cycles increases, indicating that the cyclic yield stress decreases with an increase in the number of cycles.
2. Compared to the M-C and SWT models, the improved model that accounts for mean stress effects and strength degradation demonstrates significantly higher life prediction accuracy than traditional models, with prediction errors not exceeding ±15%. Additionally, the fatigue accumulation model that considers load effects and strength degradation exhibits markedly better life prediction accuracy than the Miner and M-H models. Its fatigue damage ratios all fall within the allowable prediction error range of ±50%, and most of them can achieve a favorable prediction error within ±25%.
3. The tests revealed that the drive shaft can withstand 17,250 cycles of starting condition loads. Strain damage occurred at the junction between the relief groove and the spline on the short-shaft end spline. The error value of the improved fatigue life prediction model did not exceed ±10%. The maximum stress point on the drive shaft, as determined by the tests, exhibited the highest damage degree of 0.263 under the load spectrum. The error value of the improved fatigue cumulative damage model did not exceed ±25%. The fatigue life prediction method proposed in this study effectively enhances the accuracy of fatigue life prediction for the drive shaft.

## Figures and Tables

**Table 1:** Chemical composition of 300M steel (mass fraction %).

Element	C	Cr	Mn	Ni	Mo	S	V	Fe
Content	0.40	0.71	0.64	1.90	0.37	0.001	0.088	Bal.

**Table 2:** Torsional fatigue life of the drive shaft.

$\mathrm{Strain}\;\mathrm{Amplitude}\;\gamma_{a}$	Fatigue Life *N_f_*/Cycle	Mean Value *N_fm_*/Cycle
0.3%	10,230/10,135/10,079	10,148
0.6%	8207/8278/8232	8239
0.9%	6593/6850/6689	6710

**Table 3:** Fundamental mechanical parameters of the drive shaft under monotonic torsion at room temperature.

Parameters	Value
Shear modulus G (GPa)	0.40
Shear strength $\tau_{b}$ (MPa)	0.71
Shear strength coefficient $\tau_{0.3}$ (MPa)	0.64
Shear strength coefficient $K_{0}$	1.90
Shear strength index $n_{0}$	0.37

**Table 4:** Parameters of the mixed-hardening cyclic plastic constitutive model [23].

Parameters	Value
*C*_1_	185,648
*C*_2_	151,215
*C*_3_	33,450
*C*_4_	81,243
*C*_5_	54,652
*Q*_1_	−78
*Q*_2_	0.6546
*r*_1_	5610
*r*_2_	3458
*r*_3_	1321
*r*_4_	546
*r*_5_	125
*b*_1_	−90
*b*_2_	0.72135

**Table 5:** Torsional fatigue performance parameters.

Parameters	Value
Fatigue strength coefficient ${\tau^{\prime }}_{f}$	2024
Fatigue strength exponent b	−0.4503
Fatigue plasticity coefficient ${\gamma^{\prime }}_{f}$	2407
Fatigue plasticity coefficient c	−1.6465

**Table 6:** Life prediction results.

Materials	Test Life	Model	Prediction Life	Predicted Error
GH4133	4707	M-C [10,19]	6069	29%
		SWT [10,19]	5293	12%
		Improved model	4586	2%
Carbon steel	5640	M-C [10,19]	7177	27%
		SWT [10,19]	6542	16%
		Improved model	5035	11%
7075-T651	5275	M-C [10,19]	6956	32%
		SWT [10,19]	6254	19%
		Improved model	5725	8%

**Table 7:** Life prediction results.

Damage Degree Test Value			Damage Degree Prediction Value					
One-Level	Two-Level	Three-Level	Miner [20]		M-H [20]		Improved Model	
			Highest Damage Degree	Prediction Error	Highest Damage Degree	Prediction Error	Highest Damage Degree	Prediction Error
C35 Two-level high and low loading: $\sigma_{1}$$\;=\;353\;\mathrm{MPa},\;\sigma_{2}$ = 275 MPa								
0.10	0.45	-	0.65	44.44%	0.55	22.22%	0.37	17.78%
0.20	0.28	-	0.41	46.43%	0.38	35.71%	0.25	10.71%
0.50	0.10	-	0.14	40.00%	0.15	50.00%	0.11	10.00%
0.75	0.05	-	0.08	60.00%	0.09	80.00%	0.05	0
C35 Two-level low and high loading: $\sigma_{1}$$\;=\;294\;\mathrm{MPa},\;\sigma_{2}$ = 334 MPa								
0.10	0.86	-	0.49	43.02%	0.52	39.53%	0.69	19.77%
0.25	0.96	-	0.68	29.17%	0.77	19.79%	0.73	23.96%
0.50	0.78	-	0.29	62.82%	0.39	50.00%	0.65	16.67%
Q235 Three-level low and high loading: $\sigma_{1}$$\;=\;23.3\;\mathrm{MPa},\;\sigma_{2}$$\;=\;25.1\;\mathrm{MPa},\;\sigma_{3}$ = 26 MPa								
0.13	0.68	0.35	0.51	45.71%	0.23	34.29%	0.29	17.14%
Q235 Three-level low and high loading: $\sigma_{1}$$\;=\;38\;\mathrm{MPa},\;\sigma_{2}$$\;=\;40\;\mathrm{MPa},\;\sigma_{3}$ = 42 MPa								
0.37	0.46	0.35	0.17	51.43%	0.21	40.00%	0.39	11.43%

## Data Availability

The original contributions presented in this study are included in the article. Further inquiries can be directed to the corresponding author.

## Abbreviations

The following abbreviations are used in this manuscript:

SWT	Smith–Watson–Topper
M-H	Manson–Halford
M-C	Mason–Coffin
FEA	Finite Element Analysis
H-L	High-to-Low
L-H	Low-to-High
